# A protocol for the development of a prediction model in mild traumatic brain injury with CT scan abnormality: which patients are safe for discharge?

**DOI:** 10.1186/s41512-018-0027-4

**Published:** 2018-04-20

**Authors:** Carl Marincowitz, Fiona E. Lecky, William Townend, Victoria Allgar, Andrea Fabbri, Trevor A. Sheldon

**Affiliations:** 1 Hull York Medical School, University of Hull, Allam Medical Building, Hull, HU6 7RX UK; 2 School of Health and Related Research, University of Sheffield, Regent Court, 30 Regent Street, Sheffield, S1 4DA UK; 3 Emergency Department, Hull and East Yorkshire NHS Trust, Anlaby Road, Hull, HU3 2JZ UK; 4 Hull York Medical School, University of York, John Hughlings Jackson Building, Heslington, York, YO10 5DD UK; 5 Emergency Unit, Presidio Ospedaliero Morgagni-Pierantoni, AUSL della Romagna, via Forlanini 34, 47121 Forlì, FC Italy; 6 Department of Health Sciences, Alcuin Research Resource Centre, University of York, Heslington, York, YO10 5DD UK

**Keywords:** Mild traumatic brain injury, Prognosis, Predictive model, Intra-cranial haemorrhage, Minor head injury

## Abstract

**Background:**

Head injury is an extremely common clinical presentation to hospital emergency departments (EDs). Ninety-five percent of patients present with an initial Glasgow Coma Scale (GCS) score of 13–15, indicating a normal or near-normal conscious level. In this group, around 7% of patients have brain injuries identified by CT imaging but only 1% of patients have life-threatening brain injuries. It is unclear which brain injuries are clinically significant, so all patients with brain injuries identified by CT imaging are admitted for monitoring. If risk could be accurately determined in this group, admissions for low-risk patients could be avoided and resources could be focused on those with greater need.

This study aims to (a) estimate the proportion of GCS13–15 patients with traumatic brain injury identified by CT imaging admitted to hospital who clinically deteriorate and (b) develop a prognostic model highly sensitive to clinical deterioration which could help inform discharge decision making in the ED.

**Methods:**

A retrospective case note review of 2000 patients with an initial GCS13–15 and traumatic brain injury identified by CT imaging (2007–2017) will be completed in two English major trauma centres. The prevalence of clinically significant deterioration including death, neurosurgery, intubation, seizures or drop in GCS by more than 1 point will be estimated. Candidate prognostic factors have been identified in a previous systematic review. Multivariable logistic regression will be used to derive a prognostic model, and its sensitivity and specificity to the outcome of deterioration will be explored.

**Discussion:**

This study will potentially derive a statistical model that predicts clinically relevant deterioration and could be used to develop a clinical risk tool guiding the need for hospital admission in this group.

## Background

**Fig. 1:**
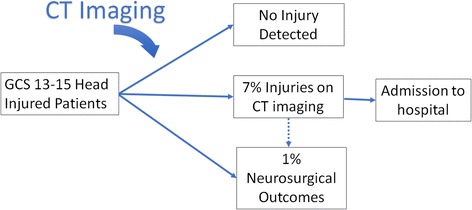
Current management of minor head injured patients

There are 1.4 million annual attendances to emergency departments in England and Wales following a head injury [1]. Approximately 95% of patients present with an initial score of 13–15 on the Glasgow Coma Scale (indicating a normal or mildly impaired conscious level) and are defined as having a “minor head injury”[2]. Minor head injured patients have a 1% risk of life-threatening traumatic brain injury (TBI) [3]. In the UK, head injury guidelines are used to triage CT imaging in this large patient population with the aim of identifying all life-threatening injuries [1, 4]. Adult guidelines are based on the internationally used and validated Canadian CT Head Rule and are applied to patients aged ≥ 16 [3, 5]. Around 7% of patients have TBI identified by CT imaging [6]. All of these patients are admitted to hospital in the UK due to fears about the risk of deterioration due primarily to intra-cranial haematoma progression, but these risks are not well characterised (Fig. 1).

The management of GCS13–15 patients with CT-identified TBI is controversial with some advocating admission to higher levels of care and mandatory repeat CT imaging due to the risk of deterioration [7]. Others argue that some patients are at low enough risk to be discharged safely from the ED after a short period of observation, a model of care adopted in a level 1 trauma centre in Arizona [8]. The UK NICE guidelines (published 2004, 2007 and 2014) state that all patients with significant brain injuries identified by CT imaging should be admitted to hospital, but do not qualify what constitutes such injuries [1].

In our recent systematic review, we estimated a pooled risk of neurosurgery in GCS13–15 patients with injuries identified by CT imaging of 3.5% (95% C.I. 2.2–4.9%) from the results of 36 studies [9]. A risk of clinical deterioration, such that patients would benefit from inpatient hospital admission, of 11.7% (95% C.I 11.7–15.8%) was derived from 18 studies. There was significant variation in estimates of these outcomes across individual studies, and no studies were conducted in the UK where NICE guidelines are used, so relevant risk factors were not considered. Following the introduction of the NICE guidelines, hospital admissions for head injury increased in England [10]. It is thought this may be due to more injuries of less clinical significance being identified due to increased CT imaging of minor head injured patients [10]. Research is required to estimate the risks of adverse outcomes in GCS13–15 patients with injuries identified by CT imaging in the UK.

GCS13–15 patients with brain injuries identified by CT imaging have a small but clinically important risk of significant adverse outcomes. Well-conducted prognostic research could generate models which allow the identification of low-risk patients who could be safely discharged from ED and high-risk patients who would benefit from more aggressive management. Our review identified 41 factors in 21 studies that had been assessed as potentially affecting the risk of adverse outcomes in this group [9]. None of this research was conducted in the UK, and no multivariable models were identified that could be used to accurately identify patients at sufficiently low risk of deterioration to be discharged from the ED. Prognostic research conducted within the context of NHS care is required to assess the extent to which GCD13–15 patients with CT-identified TBI can be stratified by risk. This will help refine the NICE guidelines and potentially allow better resource allocation in the management of these patients by identifying those who do not require hospital admission.

### Aims

1. Estimate the prevalence of clinical deterioration in initial GCS13–15 adult patients with brain injuries identified by CT imaging.
2. Develop a multivariable model that accurately identifies adult patients at sufficiently low risk of clinical deterioration that they could be discharged from the ED.

## Methods

### Study design

This is a retrospective and consecutive cohort observational study. The proportion of the cohort that clinically deteriorates will be estimated, and a multivariable prognostic model that predicts deterioration will be developed. The study will be conducted and reported in accordance with the Transparent Reporting of a multivariable prediction model for Individual Prognosis or Diagnosis (TRIPOD) recommendations [11, 12].

Patients will be identified through a retrospective case note review over a 10-year period from the end of 2007 to 2017 at Hull Royal Infirmary and Salford Royal Hospital, two English major trauma centres.

### Participants

#### Inclusion criteria

The inclusion criteria include patients aged ≥ 16 admitted to hospital, with an initial GCS of 13 or more on presentation to the ED and traumatic brain injury identified definitively by CT head imaging. All patients with epidural haemorrhage, subdural haemorrhage, subarachnoid haemorrhage, intra-cerebral haemorrhage, intra-cerebral contusion, skull fractures and any combination of these injuries will be considered for inclusion. All patients with injuries identified by CT that could only be traumatic in aetiology including skull fractures, extradural haemorrhages and subdural haemorrhages will be counted as having traumatic brain injury. Where patients have intra-cranial haemorrhage identified that could be either traumatic or spontaneous, patients will only be included if they have either a documented mechanism or evidence of head injury. This will apply to intra-cerebral and subarachnoid haemorrhages. Included mechanisms are falls, assault, road traffic collision, sport and any other mechanism that could result in blunt trauma above the clavicles. Evidence of head trauma includes bruising, wounds or injuries above the clavicles including facial and skull fractures identified radiologically.

#### Exclusion criteria

Exclusion criteria include patients with obvious penetrating head injury or with spontaneous intra-cranial haemorrhage. Patients will be categorised as having a spontaneous intra-cranial haemorrhage if the haemorrhage could occur spontaneously or traumatically and they have no documented preceding mechanism or evidence of head injury or if the CT report states that the pattern of intra-cranial haemorrhage indicates a spontaneous event. Patients with pre-existing brain injuries or other pathology that makes the interpretation of timing of injury difficult are excluded, and this includes patients with haemorrhagic brain tumours, chronic subdural haemorrhage or hygromas and other types of pre-existing intra-cranial bleeds. Patients with isolated occipital condyle fractures are excluded as these are treated as cervical spine injuries. Patients transferred from other EDs following identification of a brain injury will also be excluded.

### Study outcome

The outcome of interest is a composite measure of clinical deterioration such that inpatient hospital admission was warranted; this includes death due to TBI or neurosurgery within 30 days of attendance, ICU intervention whilst an inpatient, seizure activity whilst an inpatient, drop in GCS by 2 or more points whilst an inpatient or a readmission to hospital within 30 days of injury related to TBI.

### Candidate prognostic factors

Potential candidate factors have been selected a priori by identification of factors that individually predict deterioration in the study population in our systematic review, inclusion of additional factors that predict adverse outcomes in prognostic models for patients with more severe TBI and trauma and inclusion of factors that represent NICE guideline standards and criteria for treatment and investigation of head injury and TBI [1, 9, 13–15]. All factors being considered for inclusion in the final model are presented in Table 1 with the reason for their inclusion.

**Table 1:** Prognostic factors being investigated

Factors from systematic review	Type of data	Factors from NICE guidelines	Type of data	Factors from TARN TBI/trauma model	Type of data
Age	Continuous	1st neurological examination in ED	Categorical	Admission Hb	Continuous
Sex	Categorical	Equal pupils 1st examination	Categorical	Admission platelets	Continuous
Pre-injury anti-coagulant use	Categorical	Both pupils reactive 1st examination	Categorical	Charlson Trauma Modified Comorbidity Index	Continuous
Pre-injury anti-platelet use	Categorical	SIGN of skull fracture 1st examination	Categorical	Admission BM	Continuous
GCS on arrival to ED	Categorical	Seizures in ED	Categorical	Frailty score	Continuous
BP on arrival ED	Continuous	Vomiting in ED	Categorical		
HAIS	Continuous	An occupant ejected from a motor vehicle	Categorical		
Marshall Classification	Categorical	Mechanism of injury	Categorical		
Single injury	Categorical	Amnesia	Categorical		
Comment on midline shift	Categorical	Intoxicated EToH time of injury	Categorical		
Comment on size of bleed		Seizures before arrival ED	Categorical		
Additional injuries	Categorical	Vomiting before arrival ED	Categorical		
Sats on arrival ED	Continuous	A pedestrian or cyclist struck by a motor vehicle	Categorical		
		A fall from height of > 1 m or 5 stairs	Categorical		

Comorbidities will be measured using a trauma-modified Charlson Comorbidity Index. Brain injury severity, as shown on CT scan, will be stratified using the Marshall Classification, which will be calculated from Abbreviated Injury Severity (AIS) codes for TBI using the method described by Lesko et al. [16, 17] The Charlson Comorbidity Index, AIS and Marshall Classification are internationally validated prognostic scoring systems [18, 19]. Frailty will be assessed using the clinical frailty scale described by Rockwood et al. [20].

### Data collection

#### Screening

**Fig. 2:**
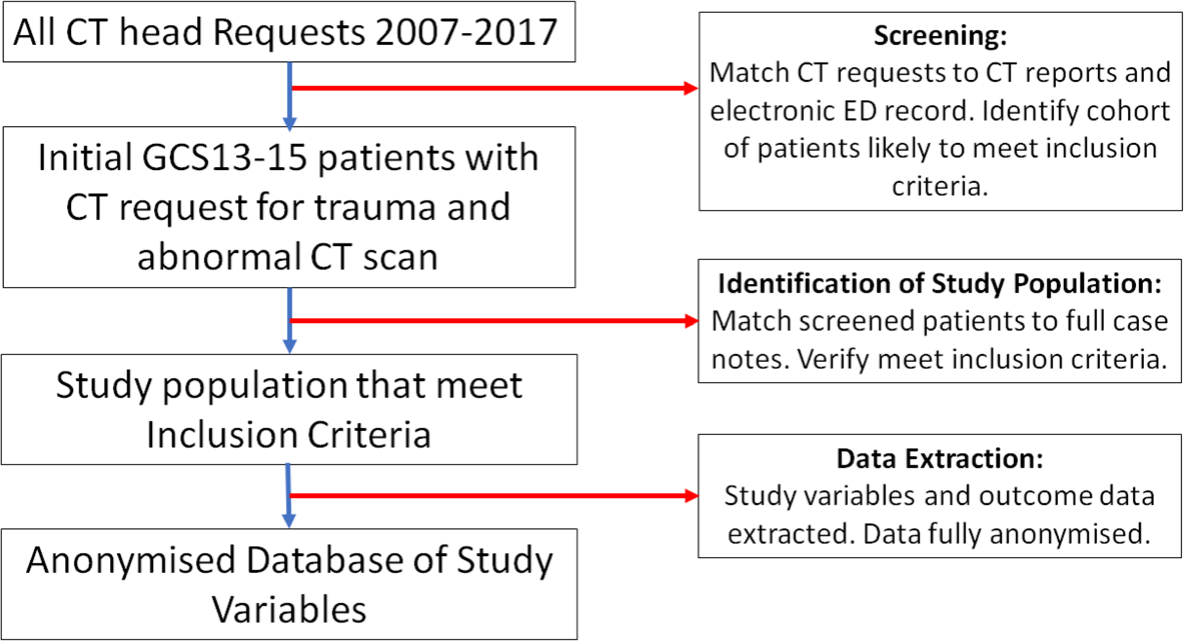
Population screening and selection

A database of all emergency department CT brain requests and reports for patients aged 16 and over between 2007 and 2017 will be generated at the two sites from the electronic requesting and reporting system. This will be screened to identify potentially eligible patients with CT requests related to head injury and CT scans with reported abnormalities related to TBI or intra-cranial haematomas (Fig. 2). Patients identified in this way will be matched to electronic ED case notes, reports and discharge summaries to identify the subset of patients potentially admitted with an initial GCS13–15.

#### Data extraction

The full case records of patients identified through screening as potentially meeting the inclusion criteria will be retrieved (Fig. 2). In patients who are confirmed to meet the inclusion criteria, all a priori candidate prognostic factors will be extracted from the case records. Demographic information will be extracted from data recorded at the time of presentation to the ED following head injury. Comorbidities, frailty and pre-injury medication use will be extracted from that recorded in the ED attendance and subsequent inpatient hospital admission documentation. Comorbidities recorded in the inpatient notes up to 1 year prior to the presentation following head trauma will be included in accordance with the method of data collection in a recent update of the Charlson Comorbidity Index [19].

The full inpatient records will be interrogated for evidence of intervention or clinical deterioration that would meet the composite outcome measure. Recorded patient ED and hospital admissions after discharge following the relevant admission for traumatic brain injury will be assessed for evidence of deterioration, intervention or readmission in the 30 days following the initial ED attendance.

Patients who were included in the national Trauma Audit and Research network (TARN) registry will be identified locally. Using an anonymous TARN study number, we will assess for any deaths recorded on the TARN registry within 30 days of admission.

#### Research team undertaking screening and data extraction

Members of the direct emergency department care team at each NHS trust will undertake the screening of electronic records for patients admitted following head injury and data extraction from case notes. Staff undertaking data extraction will undergo data extraction training, and this includes training in abbreviated injury scale coding of injuries on CT brain scans by the Trauma Audit and Research Network (TARN) which is an Association for the Advancement of Automotive Medicine-accredited trainer to ensure the use of AIS dictionary in a reliable and reproducible fashion. Data extraction will be piloted over a 1-month period. Hypothetical and non-identifiable training samples of potential patient records will be generated at both sites during the training period and will be used to check the quality of, and validate, data extraction in the research team. The research team will not be blinded to outcomes. However, most prognostic variables being collected are demographic and other factors not subject to interpretation. Patients are also not being allocated to treatment groups, and therefore, data collection is less likely to be biased in favour of a specific outcome.

### Sample size

Sample size of a prognostic study is informed by three factors: anticipated prevalence of the outcome (in this study clinical deterioration), desired sensitivity of the model to the outcome and the precision of the 95% confidence interval around the sensitivity of the model [12].

We have based our sample size on a 10% estimated prevalence of clinical deterioration in our systematic review and our desired precision of the sensitivity of the derived model for this outcome [9]. Research into discharge decision making in patients presenting to the ED with chest pain indicated that a 1/100 risk of a patient being discharged who subsequently had a significant cardiac event may be an acceptable risk threshold to both patients and clinicians [21]. Therefore, we will aim for 99% sensitivity for clinical deterioration as this may correspond to a clinically acceptable level of model accuracy.

A sample size of approximately 2000 patients is required, based upon the desired 99% sensitivity in order that the maximum marginal error of the estimate does not exceed 1.4% with a 95% confidence interval [22]. Based upon previous data collection, we estimate at least 100 patients will be eligible for inclusion per year at each site of data collection over the 10-year period of interest [23].

### Statistical analysis

#### Outcome estimate

The proportion of patients that fulfil the composite measure of deterioration will be estimated. A sample size of 2000 patients will allow us to estimate the prevalence of clinically significant deterioration with a 1.3% margin of error at a 95% confidence level.

#### Model development

Multivariable logistic regression with backward stepwise selection will be used to find the best combinations of candidate factors highly sensitive for detecting deterioration while achieving the maximum possible specificity. This approach is favoured as all correlations between predictors are considered in the modelling procedure and there is easier transparency of reporting [12].

Candidate prognostic factors with a *P* value greater than 0.05 will be selected for removal. Forced variables (predictors) that we consider as having clinical relevance, as indicated in our systematic review and the NICE guidelines, will initially also be considered for inclusion in our model and retained in the initial steps of backwards elimination. In the final model, all factors that do meet the significance level will be removed.

The sample size of 2000, with an anticipated prevalence of clinical deterioration of around 10%, will allow the model to include 20 variables, based on the rule of at least 10 outcome events per parameter estimated.

Continuous factors will not be categorised initially to avoid a loss of power [24, 25]. Calibration (the agreement between outcome predictions from the model and the observed outcomes) will be tested with the Hosmer–Lemeshow test. We will assess the apparent performance of the fitted models for discrimination using the C-statistic (equal to the area under the receiver operating characteristic curve) and the sensitivity for clinically significant deterioration [26].

Internal validation using the bootstrap validation approach will be undertaken to evaluate the performance and optimism of the developed model [27]. This will allow the use of the complete data set for model development and provide a mechanism to account for model overfitting or uncertainty in the model development process. We will quantify any optimism in the final prediction model and estimate a so-called shrinkage factor that can be used to adjust the regression coefficients and apparent performance for optimism. This will lead to a new final model being produced in each of the bootstrap samples. We will average the difference in the performance of the models to obtain a single estimate of optimism for the C-statistic.

#### Missing data

As data are to be extracted from clinical records, missing variable data will inevitably occur. Although it is possible to verify the data to judge whether missing data are missing completely at random (MAR) or associated with observed variables, it is generally impossible to prove that data are indeed MAR or whether they are not missing at random (MNAR) [24]. Multiple imputation will be used to impute missing data with the number of imputations determined by the amount of missing data, under a missing random assumption, so as to avoid excluding patients from the analysis. This will be completed using STATA with the exact method determined by the amount, type and distribution of the missing data, and we will adhere to recognised guidelines for appropriate use and reporting of methods to deal with missing data [28, 29]. After imputation, a sensitivity analysis will be undertaken to determine how the substantive results depend on the multiple imputation method employed. This is consistent with the TRIPOD recommendations with the handling of missing data in prognostic studies [12].

#### Model accuracy

The sensitivity and specificity of the model for detecting patients at low risk of deterioration will be calculated comparing the classification of each patient by the model with whether they actually deteriorated. To assess how informative lack of deterioration is, the model will be derived again for those patients who do not deteriorate within 24 h. We will determine whether a more accurate model can be produced for those still in hospital after 24 h.

A receiver operating characteristic (ROC) curve for both models will be plotted and the trade-off between the sensitivity and specificity of the model explored [30]. As indicated previously, a 1/100 risk of deterioration following discharge may be clinically acceptable, and therefore, our model will aim for at least a 99% sensitivity to deterioration [21].

#### Sensitivity analysis

The 10-year period of data collection represents a long time period over which clinical practice and outcomes may have changed. To assess for this, we will estimate the yearly prevalence of clinical deterioration and note any statistically significant changes in outcome over time. In addition, because NICE guidelines were updated in 2014 (with minor changes to the indications for CT brain imaging), the prognostic model will be estimated solely for the time period 2014–2017 and compared to the model estimated for the whole time period [1].

#### Exploratory analysis

Individual patient data from a prospective Italian cohort study is available to the research team [31]. The variables collected in the Italian study and how they compare to the variables being collected in our study are shown in Table 2. If most factors present in the multivariable model developed in our study are present in the Italian data set, then we will assess the effect of these factors on the risk of deterioration in a multivariable model derived in the Italian data set. If the effect estimates are similar to those estimated in the data collected in England, then we will combine the individual patient data of the two data sets to improve the precision of the model estimates.

**Table 2:** Comparison between Italian data set and data being collected

Factor	In Italian data	Factor	In Italian data
Age	Yes	Equal pupils 1st examination	Yes
Sex	Yes	Both pupils reactive 1st examination	Yes
Pre-injury anti-coagulant use	Yes	SIGN of skull fracture 1st examination	No
Pre-injury anti-platelet use	No	Seizures in ED	No
Charlson Trauma Modified Comorbidity Index	Yes	Vomiting in ED	No
A pedestrian or cyclist struck by a motor vehicle	Yes	HAIS	No
An occupant ejected from a motor vehicle	Yes	Marshall Classification	Yes
A fall from height of > 1 m or 5 stairs	Yes	Single injury and type of injury	Yes
Mechanism of injury	No	Comment on midline shift	No
Amnesia	Yes	Comment on size of bleed	No
Loss of consciousness	Yes	Frailty score	No
Intoxicated time of injury	No	Admission Hb	No
Seizures before arrival ED	Yes	Admission platelets	No
Vomiting before arrival ED	Yes	Admission BM	No
GCS on arrival to ED	Yes	Additional injuries	Yes
BP on arrival ED	No		

## Discussion

### Strengths

To the authors’ knowledge, this will be the largest cohort study conducted that assesses clinical deterioration in GCS13–15 patients with brain injuries identified by CT imaging. We are collecting data from multiple sites and potentially incorporating data from a different European country. The definition of clinical deterioration is wide and defined to encompass potential benefits of hospital admission from the ED. This outcome is one that can be used to help inform clinical decision making regarding the selection of patients in this group that would benefit from hospital admission.

### Limitations

Data collection is retrospective and will be limited by the nature and accuracy of the data clinically recorded. However, such data are likely to be applicable and implementable in current routine practice. Given the large sample size required for this study and the challenges of prospectively recruiting patients in the ED, a retrospective method for data collection represents a feasible and pragmatic data collection strategy.

Outcomes will only be assessed during hospital admission and re-attendances to the hospitals where data collection is occuring. This may underestimate deterioration following discharge especially if patients die in the community or deteriorate and are readmitted to a different hospital. We will estimate the effect of this possible bias by conducting a sensitivity analysis using data for the subset of patients registered on the Trauma and Audit Network Database where complete data following discharge is available.

### Further research

Prognostic models tend to perform optimistically using the data from which they were derived, and therefore, their accuracy requires external validation in separate data sets [12]. There are different strategies for this and we will attempt to validate the model derived from this study in a sub-population of a European prospective cohort of TBI patients that is currently ongoing (CENTER-TBI), with data expected to be available in 2018 [32, 33]. Our validation study will be subject to a separate protocol. If the model appears sufficiently accurate at identifying low-risk TBI, that patients could be safely discharged, implementation will be tested prospectively in the context of the NHS.

## Abbreviations

AIS: Abbreviated Injury Severity
CT: Computed tomography
GCS: Glasgow Coma Scale
NICE: National Institute for Health and Clinical Excellence
ROC: Receiver operating characteristic
TARN: Trauma Audit and Research Network
TBI: Traumatic brain injury

## References

[1] NICE, National Clinical Guidance Centre. (2014). CG 176 head injury triage, assessment, investigation and early management of head injury in children, young people and adults. National Institute for Health and Care Excellence., NICE, Editor. 2014, DOH: UK. 25340248

[2] Miller JD (1986). Minor, moderate and severe head injury. Neurosurg Rev.

[3] Stiell IG (2001). The Canadian CT Head Rule for patients with minor head injury. Lancet.

[4] Network, S.I.G (2009). Guideline 110: early management of patients with a head injury.

[5] Smits M (2005). External validation of the Canadian CT Head Rule and the New Orleans Criteria for CT scanning in patients with minor head injury. JAMA.

[6] Haydel MJ (2000). Indications for computed tomography in patients with minor head injury. N Engl J Med.

[7] Thorson CM (2013). Repeat head computed tomography after minimal brain injury identifies the need for craniotomy in the absence of neurologic change. Journal of Trauma & Acute Care Surgery.

[8] Joseph B (2014). The BIG (brain injury guidelines) project: defining the management of traumatic brain injury by acute care surgeons. J Trauma Acute Care Surg.

[9] Marincowitz C (2018). The risk of deterioration in GCS13-15 patients with traumatic brain injury identified by computed tomography imaging: a systematic review and meta-analysis. J Neurotrauma.

[10] Goodacre S (2008). Hospital admissions with head injury following publication of NICE guidance. Emerg Med J.

[11] Royston P (2009). Prognosis and prognostic research: developing a prognostic model. BMJ.

[12] Moons KG (2015). Transparent Reporting of a multivariable prediction model for Individual Prognosis or Diagnosis (TRIPOD): explanation and elaboration. Ann Intern Med.

[13] Steyerberg EW (2008). Predicting outcome after traumatic brain injury: development and international validation of prognostic scores based on admission characteristics. PLoS Med.

[14] Roozenbeek B (2012). Prediction of outcome after moderate and severe traumatic brain injury: external validation of the International Mission on Prognosis and Analysis of Clinical Trials (IMPACT) and Corticoid Randomisation After Significant Head injury (CRASH) prognostic models. Crit Care Med.

[15] Lesko MM (2013). Models of mortality probability in severe traumatic brain injury: results of the modelling by the UK trauma registry. J Neurotrauma.

[16] Lesko MM (2010). Using Abbreviated Injury Scale (AIS) codes to classify Computed Tomography (CT) features in the Marshall System. BMC Med Res Methodol.

[17] Bouamra O (2015). Prediction modelling for trauma using comorbidity and ‘true’ 30-day outcome. Emerg Med J.

[18] Raabe A (1998). Correlation of computed tomography findings and serum brain damage markers following severe head injury. Acta Neurochir.

[19] Quan H (2011). Updating and validating the Charlson comorbidity index and score for risk adjustment in hospital discharge abstracts using data from 6 countries. Am J Epidemiol.

[20] Rockwood K (2005). A global clinical measure of fitness and frailty in elderly people. CMAJ.

[21] Hess EP (2016). Shared decision making in patients with low risk chest pain: prospective randomized pragmatic trial. BMJ.

[22] Hajian-Tilaki K (2014). Sample size estimation in diagnostic test studies of biomedical informatics. J Biomed Inform.

[23] Marincowitz C, Allgar V, Townend W (2016). CT head imaging in patients with head injury who present after 24 h of injury: a retrospective cohort study. Emerg Med J.

[24] Little RJA RD (2002). *Statistical analysis with missing data*.

[25] Royston P, Sauerbrei W (2005). Building multivariable regression models with continuous covariates in clinical epidemiology with an emphasis on fractional polynomials. Methods Inf Med.

[26] Steyerberg EW, Vergouwe Y (2014). Towards better clinical prediction models: seven steps for development and an ABCD for validation. Eur Heart J.

[27] Harrell FE, Lee KL, Mark DB (1996). *Multivariable prognostic models: issues in developing models, evaluating assumptions and adequacy, and measuring and reducing errors*. Stat Med.

[28] Royston P (2005). Multiple imputation of missing values: update. Stata J.

[29] Sterne JA (2009). Multiple imputation for missing data in epidemiological and clinical research: potential and pitfalls. BMJ.

[30] Florkowski CM (2008). Sensitivity, specificity, receiver-operating characteristic (ROC) curves and likelihood ratios: communicating the performance of diagnostic tests. Clin Biochem Rev.

[31] Fabbri A (2008). Observational approach to subjects with mild-to-moderate head injury and initial non-neurosurgical lesions. J Neurol Neurosurg Psychiatry.

[32] Maas AI (2015). Collaborative European NeuroTrauma Effectiveness Research in Traumatic Brain Injury (CENTER-TBI): a prospective longitudinal observational study. Neurosurgery.

[33] Altman DG (2009). Prognosis and prognostic research: validating a prognostic model. BMJ.

